# What regenerative agriculture can teach medical students about human health

**DOI:** 10.5116/ijme.6463.4962

**Published:** 2023-05-30

**Authors:** David Ebbott, Dimitrios Papanagnou

**Affiliations:** 1 Sidney Kimmel Medical College at Thomas Jefferson University in Philadelphia, PA, USA; 2 Department of Emergency Medicine, Sidney Kimmel Medical College at Thomas Jefferson University, Philadelphia, PA, USA

**Keywords:** Regenerative agriculture, teaching, medical students, human health

## To the Editor

The medical and agricultural communities have supported a paradigm shift for thinking more about food as medicine. Recent epidemiologic trends show continued rises in non-communicable inflammatory diseases (NCDs), such as heart disease, diabetes, obesity, malignancies, and mental health disorders. The number of studies connecting NCDs with environmental and ecological factors has increased dramatically.^1^ From a medical standpoint, burgeoning research includes the complex interplay of nutrition, gut microbiomes, and inflammatory modulation.^2^^,^^3^ From an agricultural standpoint, research has included the study of regenerative agriculture, specifically examining the interplay of soil biodiversity, nutrient exchange, and crop nutrient-density.^4^^,^^5^ Regenerative farms employ agricultural principles that protect soil health to support ecosystem and crop health sustainability. The medical and agricultural evidence has supported robust and increasingly impactful discussions surrounding food as medicine. Specifically, research has explored the soil-gut connection, examining the link between biodiversity at the crop-soil level for nutrient density and its impact on the human-gut microbiome and its downstream inflammatory consequences.^5^^,^^6^ As Hippocrates said, “all diseases start in the gut.” We can trace this statement one step further by considering the direct source of our nutrients, and posit that many diseases potentially have origins in soil.

Considering the standard, the post-Flexner paradigm of medical education that aligns physician professional roles and respective competencies with the current and future needs of patients, health systems, and society,^7^ students typically learn to guide their patients through exacerbations of inflammatory conditions during their clinical clerkships. In this educational context, students learn to counter excessive inflammation and disrupted metabolism with immune modulators and resection of diseased tissue. But is there an opportunity to prepare students to understand the roots of these chronic epidemic diseases and better prepare them to address these diseases from the ground up, literally? We believe there are forward-thinking educational opportunities that have the potential to recalibrate student perceptions of food as medicine.

Consider an immersive educational experience (i.e., elective, course, extracurricular rotation) that positions a medical student to work and study on a regenerative farm. Students will have the unique experience of joining other regenerative and/or permaculture interns as they practice nurturing plants, crops, and animals to harness the biodiversity of the soil for sustainable nutrient exchange between the soil and crops. They will also have the opportunity to explore topics that sit at the intersection of crops and human health, such as soil health, soil microbiomes, nutrient exchange and nutrient density, crop health, human microbiomes, human health, soil erosion, chemical runoff, and carbon sequestration.

We hypothesize layered benefits of these educational experiences – experiences that have the potential to expand students’ skillsets relative to providing care that is individually based, but also population-minded and future-oriented. First and foremost, students will gain first-hand knowledge of what is meant by food as medicine. This would encompass a different perspective on nutrient exchange and how this impacts the biodiversity in our own bodies. It will transcend students beyond the necessary, but at times esoteric, dry diagrams of nutrition to authentically witness the role balanced, well-sourced crops play in nutrient cycling. Giving students an active role in ecological mitigation efforts may also increase their long-term interest in the environmental determinants of health. At the highest level, students will practice tracing epidemiologic chronic health problems to their origins. In an age of climate change and resource scarcity, students will require the background knowledge to think critically about nutrition-related problems of tomorrow.

It is expected that skills gained in these field experiences would influence performance in the clinical setting. For example, students may feel more confident when counseling patients on nutrition for issues ranging from constipation to preventing autoimmune exacerbations. Students may also gain a broader perspective for performing clinical assessments and development of their patient management plans. From the lens of research, students may develop an enhanced interest in nutrition or how biodiversity within the human taxa impacts health. In public health settings, students may give heightened attention to disease-modifying environmental interventions. As an unintended byproduct of this educational innovation, students may feel reduced burnout with the wellbeing and rejuvenation associated with being immersed in a natural setting for a short segment of their formal training.

The educational value of working and studying on a regenerative farm is naturally rooted in adult learning theory. At its core, the learning that would take place is experiential and constructivist in nature, with students practicing environmental and human health interventions in a live, organic setting. Using all five senses in such a setting, combined with reflective discourse, will allow students to make meaningful, lasting connections between the natural world, nutrition, and human health.

Naturally, there are several barriers to implementation that merit consideration, particularly prioritizing cross-discipline educational experiences and developing lasting partnerships with local farms. Given the need for medical schools to expand the breadth of educational offerings in the face of increased student learners, however, an educational experience in regenerative farming can complement pre-clinical and clinical content while infusing subject matter with the social sciences. Furthermore, there is the untapped opportunity to cultivate relationships with famers who are willing to volunteer and offer agricultural instruction to further amplify the importance of this field with the greater professional community.

In closing, curricular interventions that leverage immersive experiences in regenerative agriculture can provide students with a clinical and public health understanding of food as medicine, as well as an understanding of nutrient exchange, microbiome-linked ecological principles, and the environmental determinants of health. The inflammation that has spread across populations at epidemic levels has direct ties to how our societies grow food, obtain nutrients, and support the internal human ecosystem. Through the biological and social aspects of regenerative agriculture, educators have the opportunity to assist students with considering diseases at their roots and appreciating the therapeutic benefits of viewing food as medicine. Collectively, the addition of this content into formal curriculum has the novel potential to help students trace sources of epidemic-level NCDs from soil to crop to gut, and better position them to address them with their patients, their communities, and their research stakeholders.

### Conflict of Interest

The authors declare that they have no conflict of interest.
